# Effects of an odor or taste stimulus applied to an artificial teat on the suckling behavior of newborn dairy calves

**DOI:** 10.1186/s40781-018-0164-x

**Published:** 2018-04-16

**Authors:** Maria Malidaki, Matthias Laska

**Affiliations:** Department of Physics, Chemistry and Biology, Linköping University, SE-581 83 Linköping, Sweden

**Keywords:** Artificial teat, Dairy calves, Glucose, Odor, Suckling behavior, Taste

## Abstract

**Background:**

In their first days of life, dairy calves in artificial rearing systems often have difficulty using an artificial teat for feeding.

**Methods:**

We examined the age at which calves are able to stand up voluntarily and suckle as well as their suckling behavior when presented with a plain dry teat versus a dry teat modified with a presumably attractive odor or taste substance. Single-housed newborn dairy calves (*n* = 51) were presented for ten consecutive days with a two-minute two-choice test, in which suckling time was recorded for 1) a plain (control) teat versus a glucose-coated teat (taste test) and 2) a plain teat versus a teat with a "Freshly Cut Grass" odor (odor test).

**Results:**

On average, the calves were able to stand up voluntarily and suckle from the second or third day of age on. The "Freshly Cut Grass" odor had no significant effect on their suckling behavior. In contrast, the calves showed a significant preference for suckling the glucose-coated teat and displayed a significantly longer total suckling time in the taste test compared to the odor test. There were no significant differences between sexes regarding suckling behavior.

**Conclusion:**

The results of the present study show that glucose had a significant effect on the calves’ teat preference and significantly increased total suckling time with a dry artificial teat. As such, glucose may increase suckling motivation in non-efficient drinkers or ill calves with low motivation to suckle.

**Electronic supplementary material:**

The online version of this article (10.1186/s40781-018-0164-x) contains supplementary material, which is available to authorized users.

## Background

It is common practice for commercial dairy farms to separate the newborn calf from the dam immediately or within a few hours after birth. When allowed to stay with the dam, the calf will use several natural cues (e.g. the dam’s shape, odors, vocalizations and cow-calf interactions such as the dam licking the calf) to locate the udder and attempt to suckle [1–3]. However, even in the presence of the dam, successful suckling is not always guaranteed [3–5]. Thus, newborn calves usually require assistance from the farm staff during their first suckling efforts [6]. One of the main tasks for the caretakers is to introduce the calves to suckling from an artificial teat attached to a milk bucket (teat bucket) [1].

To our knowledge, only a few studies have investigated some aspects of suckling behavior in artificially reared calves during the very first days of life. Stafford et al. [7] reported that, on average, healthy newborn calves learn to suckle from an artificial teat within the first two days of their lives. Some calves can be more successful than others in this procedure, but calves that have problems suckling from an artificial teat can miss a number of meals before they succeed in using the artificial teat without assistance from the staff [7]. Loberg and Lidfors [8] studied the effects of providing an artificial teat versus an open bucket on the cross-suckling behavior between dairy calves as young as four days of age, while Hänninen et al. [9] investigated the effects of different colostrum feeding methods (suckling the dam, suckling from an artificial teat or drinking from an open bucket) on the suckling and resting behavior of two- and three-day-old calves; however, none of these studies make mention of the efficiency of newborn calves in using an artificial teat.

Several factors may hinder an otherwise healthy newborn calf from accepting the artificial teat. With the dam removed, the single calf pens lack the sensory cues a calf would encounter under natural circumstances in order to stand up and suckle [1, 2, 10]. The artificial teat, usually made of silicon or latex, might not be as attractive to the calf as the natural teat due to differences in e.g. texture, firmness, temperature or odor. Separation stress [11–13] may also be a hindrance, particularly for calves that spent several hours with their dam before being separated and transferred to a single calf pen.

The events taking place during the first days of a calf’s life are crucial to its survival, and proper nutrition is one of the most important factors for keeping the calf alive and healthy. Aside from the welfare considerations of hunger-induced stress [14, 15], lack of nutrients due to undernutrition may compromise the calf’s immune system, leaving it more susceptible to neonatal diseases [16]. Furthermore, calf caretakers are required to spend a substantial amount of time helping the problematic newborn calves to suckle, which can lead to disruption of the frequently intense farm routines.

In the present study, we examined the possibility of stimulating newborn calves that are efficient drinkers to suckle an artificial teat that did not provide a milk reward (dry teat) by using a potentially attractive odor or taste substance, respectively, as sensory stimuli that can be applied near or on the teat. We assessed: a) the age at which newborn calves could suckle from an artificial teat by standing voluntarily without assistance, b) whether newborn calves showed a preference for suckling a plain artificial teat versus an artificial teat modified with an odor or a taste substance, c) whether male and female calves differed in their suckling behavior, and finally d) whether a taste or an odor stimulus was more effective in stimulating the calf’s motivation to suckle. Identifying a potential preference for a substance in efficient drinkers could permit further investigation on the effects of this substance in the suckling motivation of non-efficient drinkers.

## Methods

### Location and animal management

The study was conducted at Vasen Dairy Farm in Nye, Jönköping county, Sweden, between June and August 2016. The farm maintains approximately 450 dairy cows, mainly Swedish Holstein and Swedish Red breeds, with an average rate of 50 births per month. In accordance with the management of newborn animals at the farm, the calves were separated from the dam between the first and the third day of age. Before transfer to single pens, the calves were fed by suckling the dam and by using a calf bottle with a rubber teat two to three times per day. Calves in the single pens were fed milk twice per day, at 7:00 h and at 16:00 h, suckling from a teat bucket. The teat buckets were removed after each feeding session.

### Testing method

Testing was conducted daily at the single calf pens between 12:00 and 15:00 h, for a total of 10 consecutive days per calf. Calves transferred to the single pens on the morning of a testing day received a brief health check from a veterinarian. Calves requiring veterinary treatment on the day of transfer were excluded from testing. The calves were then assessed for their motivation to stand up voluntarily and suckle in the single calf pen by stimulating the suckling reflex. This was achieved by rubbing a finger against the calf’s lips or by inserting a finger in the calf’s mouth for a few seconds. Calves that suckled the finger, subsequently stood up voluntarily without assistance and continued suckling or sought to suckle were marked as “motivated”. The rest of the calves were marked as “unmotivated”. “Motivated” calves were assigned to the taste and odor tests, while “unmotivated” calves were assigned to the suckling reflex test, as described below. All “unmotivated” calves were controlled for their ability to stand and for body temperature before their participation in the suckling reflex test; all of the animals could stand without assistance and had regular body temperature (38.6–39.4 °C) on the testing day.

### Taste and odor tests

The taste and odor tests were conducted once per day for each calf. “Motivated” calves were tested for ten consecutive days (testing sessions 1–10), while “unmotivated” calves were tested for nine consecutive days (testing sessions 2–10) after having participated in the suckling reflex test on day one. These tests were performed in order to assess whether the calves showed a preference for suckling a plain (control) dry teat versus a dry teat that was modified with a presumably attractive taste or odor. Calves that required veterinary treatment were excluded from testing on that testing day, and the testing session was marked as "did not participate".

**Fig. 1:**
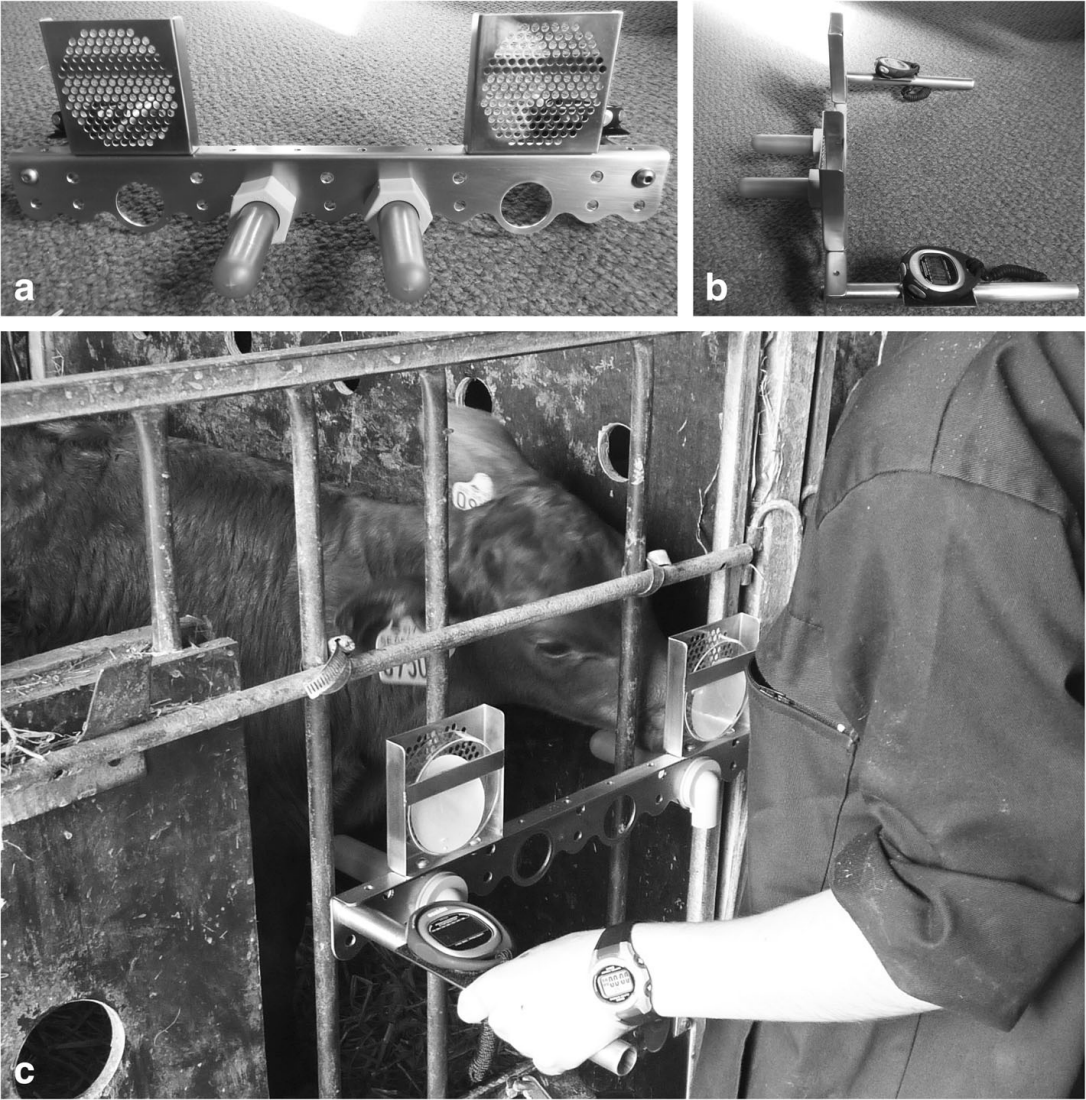
Two-choice test apparatus for the taste and odor tests. **a**. Front view, with the teats in position for the taste test; **b**. Side view, with the teats in position for the taste test; C. Example of apparatus positioning during testing, with the teats in position for the odor test

A two-choice test apparatus was used that could be adapted to the requirements of each test and that allowed the simultaneous presentation of the control and the modified teat to the calf (Fig. 1). The apparatus was made of stainless steel and consisted of:

- An “inverted L-shaped” bar (50 × 2 × 6.5 cm, 2 mm thick), with four holes of 3.8 cm diameter each. The adjacent holes had a distance of 10 cm from one another, measured at the center of each hole. The two centermost holes were used for positioning the teats for the taste test. The two outermost holes were used for positioning the teats for the odor test. Thus, the teats for the taste test had a distance of 10 cm from one another, allowing the calf to perceive and taste both with ease. The teats for the odor test had a distance of 30 cm from one another. This distance created sufficient space between the odor stimulus placed above one of the teats (as described below) and the blank stimulus placed above the other teat in order for them to be distinguishable, and at the same time allowed the calf to perceive both teats.
- Two mesh cases (9.5 × 2.3 × 9.5 cm, 1 mm thick) with holes of 5 mm diameter. The mesh cases were placed above the outermost holes of the metal bar where the teats for the odor test were positioned. Open petri dishes containing odorized or non-odorized filter papers were inserted in the mesh cases, with the open side facing the mesh for stimulus dispersal towards the teat (as shown in Fig. 1, Panel c).
- Two cylindrical handles (3.9 × 25 cm) positioned at the outermost parts of the “inverted L-shaped” bar used by the experimenter to hold the apparatus. Each handle bore a rectangular surface (7 × 5 cm) allowing the attachment of a stopwatch. The stopwatches were used for measuring suckling time for each teat.

For the *taste test*, the modified teat consisted of a plain teat dipped in water and then coated with glucose ("Just 100% Dextro", 13: e Protein Import AB, Skogås, Sweden), using a shaker to apply approximately 0.70 g of the substance as evenly as possible on the teat surface. Glucose was selected as the presumably attractive taste stimulus according to the findings of Hellekant et al. [17] regarding behavioral responses of calves to sweeteners, and due to the frequent availability of the substance in dairy farms for the preparation of electrolyte solutions for calves.

It has been clearly demonstrated that the taste of milk elicits suckling in calves of at least three weeks of age [18–20], who should by that time be fully efficient in suckling from an artificial teat. As our testing group for the current study involved efficient drinkers, we opted against using milk as a presumably attractive substance due to its known effects on such a group. However, to our knowledge, it has not been investigated if the taste and smell of milk on the artificial teat have similar effects on the suckling motivation of newborn dairy calves immediately after birth and up until the point where they become efficient drinkers a few days later. In October 2017 we conducted a preliminary online survey asking dairy calf caretakers in Sweden about newborn calves having difficulty using the artificial teat on the first 24–72 h after birth. The responses of the survey, available in the supplementary material of this study [see Additional file 1], suggest that the taste of milk alone is not always successful in motivating newborn dairy calves to use the artificial teat. For this reason, it would be beneficial to initially investigate the effects of other potentially attractive substances that imitate or belong to the repertoire of tastes and odors that a calf encounters either during gestation or during the early days of life.

For the *odor test*, the modified teat consisted of a plain teat with an open petri dish positioned vertically above it which contained a filter paper impregnated with 1 mL of the odor "Freshly Cut Grass Fragrance Oil" (Mystic Moments, Alderholt, UK). The odor was selected as a presumably attractive olfactory stimulus according to the fact that grass is an important part of cattle diet [3, 21] and volatile compounds from grass feed are transferred to milk [22–24] that calves consume at least during the first days of life. As control odor, a petri dish containing a filter paper impregnated with 1 mL of water was placed above the plain (control) teat. The petri dishes were secured behind a mesh, so that the calves could smell but not come in direct physical contact with or lick the substances (see Fig. 1).

The majority of the calves were tested while standing and facing the experimenter, with the apparatus held at the head level, ensuring equal ease of access to both teats (Fig. 1, Panel c). On the few occasions that a calf was unable to or refused to stand, it was tested while lying on its chest and facing the experimenter. The calf was then allowed to interact with the teats for a period of 2 min. The rationale for using this period of time was that Ventorp and Michanek [25] defined a period of at least 0.5 min as successful suckling in calves.

The following data were recorded for each individual and test session:

- investigating behavior consisting of sniffing, touching with the lips or licking the tip of a teat, or immediately suckling a teat,
- order of teat investigation, i.e. which of the teats was investigated first (control teat first / modified teat first / did not investigate the teats), and
- suckling time for each teat, in seconds, with the use of stopwatches.

Four latex rubber teats were used throughout the study (Foga Försäljning AB, Sweden). During a single testing day, one pair of teats was used for all the taste tests and one pair was used for all the odor tests, with each teat being randomly assigned the role of control or modified teat throughout the testing day. In order to avoid preference bias due to predictable teat placement (right or left side), the positioning of the control and the modified teat was pseudo-randomized throughout the testing days, but was always opposite for the two tests on a given testing day (e.g. modified teat positioned right for the taste test, and left for the odor test). After testing a calf, each teat was rinsed with water and, if necessary, wiped with a paper towel before being used for the next calf.

On each testing day, all calves were first tested with the taste test in order to avoid potential contamination of the apparatus from the test odor. Before conducting the odor test, the apparatus was wiped with a wet paper towel or, if necessary, rinsed with water, in order to remove potential glucose remnants. The time interval between the two tests for a single calf was at least five minutes.

At the end of each testing day, the apparatus and the teats were thoroughly cleaned with hot water and fragrance-free soap and were left to dry until the next day.

### Suckling reflex test

The suckling reflex test was conducted only during the first testing day of “unmotivated” calves in order to assess whether a calf would successfully suckle a plain teat as well as the modified teats (taste and odor) described in the previous section. The same apparatus (Fig. 1) was used for this test. During the test, a plain teat was first presented to the calf in front of its muzzle, then inserted into the calf’s mouth for approximately five seconds, and the suckling response was recorded (suckled / did not suckle). The same procedure was repeated with a glucose-coated teat. For the teat that was modified with an odor, the calf was first presented with the "Freshly Cut Grass" odor by placing the mesh of the test apparatus bearing the odor stimulus directly in front of its muzzle and then the teat was inserted into the calf’s mouth, following the same procedure as with the other two teats. A “licking lips” behavioral response was also recorded during the presentation of each teat to the calf (performed / not performed). The behavior consisted of the calf licking the lower part of its muzzle, with the tongue being visible, for approximately one to three repetitions.

### Sample size and age

**Fig. 2:**
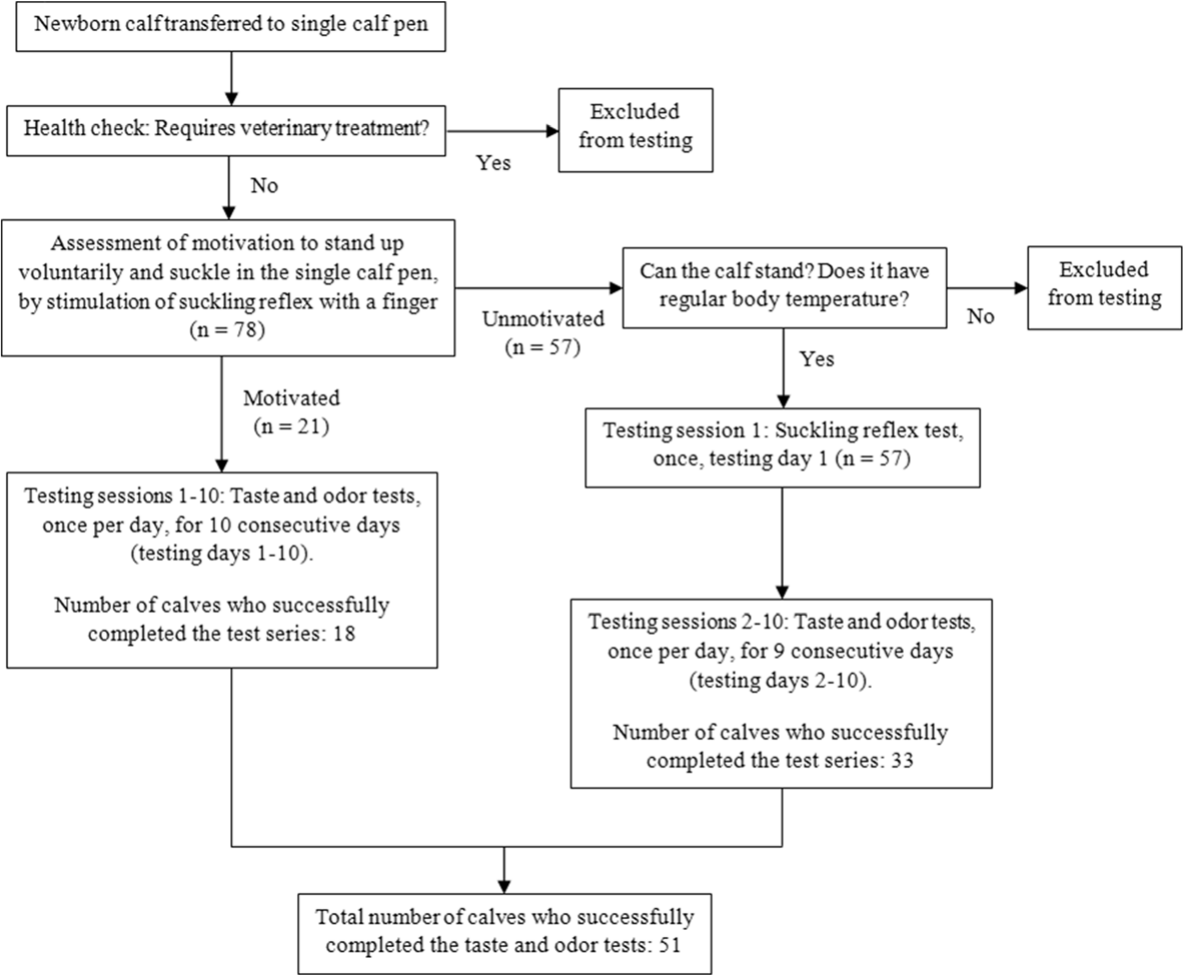
Flow chart representing the flow of events throughout the study. The criteria used for the inclusion of a calf into the study are also shown

In total, 78 newborn dairy calves (*n* = 78) were sampled for the study. Fifty-seven of these calves (*n* = 57) were marked “unmotivated” on the first day of testing and thus successfully participated in the suckling reflex test. Fifty-one out of 78 calves (*n* = 51), of which 29 male and 22 female, successfully completed the series of taste and odor tests. Successful completion of the taste and odor tests was achieved if a calf successfully participated in at least six out of nine tests (“unmotivated” calves) or seven out of ten tests (“motivated” calves), for the taste and the odor test, respectively. Participation was considered successful if a calf suckled at least one of the two teats for at least one second. The age of the calves when entering the testing group varied between one and five days old. Thus, all measurements took place within the calves’ first two weeks of age. Figure 2 summarizes the criteria used for inclusion of a calf into the study and the flow of events throughout the study.

### Data analysis

The Chi-squared test was used to assess whether the “licking lips” behavior was performed by a significant proportion of calves during the suckling reflex test. The two-tailed binomial test was used to assess whether the calves showed a significant preference for the order of investigation of a teat (control or modified) during the odor test and the taste test. The Wilcoxon signed-ranks test for related samples was used to assess if there were significant differences between the suckling time for the control teat (ST_C_) and the suckling time for the modified teat (ST_M_) in the odor test and the taste test, as well as between total suckling times (ST_T_ = ST_C_ + ST_M_) for the odor test versus the taste test. For this statistical test, an average suckling time per calf for each type of teat or test was first calculated (sum of suckling times per teat for all testing sessions of an individual, divided by the number of testing sessions, when comparing the control teat and the modified teat; sum of ST_T_ for all testing sessions of an individual, divided by the number of testing sessions, when comparing the odor test and the taste test). The Mann-Whitney U-test for independent samples was used to assess if there were significant differences regarding suckling times between male and female calves. For this statistical test, an average suckling time for each type of teat (odor, taste) was calculated for each calf, and the sum of average suckling times was used to calculate each sex average. All statistical analyses were performed using the software R (version 3.3.1, ran of R Studio environment version 0.99.903).

## Results

### Use of the artificial teat in relation to age

**Fig. 3:**
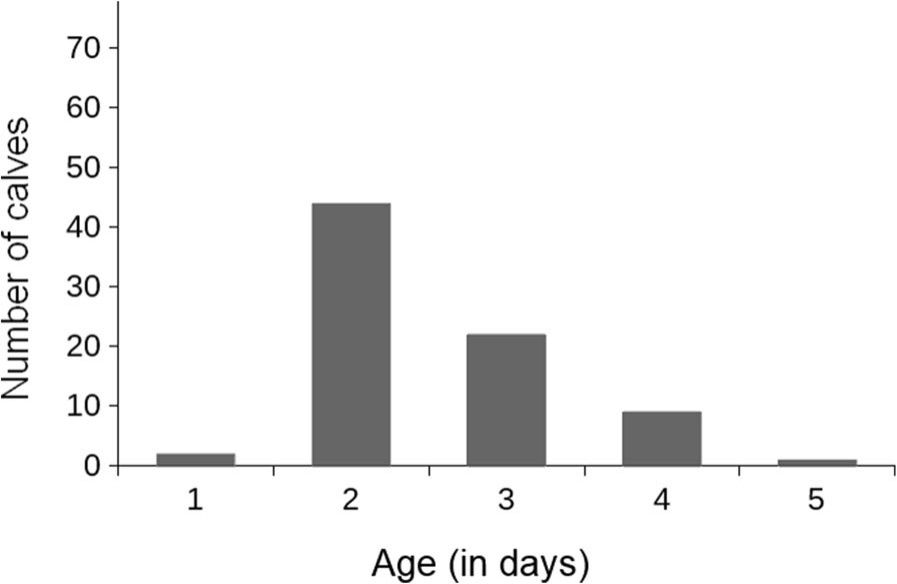
Use of the artificial teat in relation to age. The diagram shows the age at which calves (*n* = 78) were able to stand up voluntarily and suckle an artificial teat for the first time

The age at which the newborn calves of this study were able to use the artificial teat by standing voluntarily without assistance varied between one and five days. The majority of the calves were able to stand up voluntarily without assistance and suckle the artificial teat for the first time on the second (44 calves out of 78) or the third (22 calves out of 78) day of age (Fig. 3).

### Suckling reflex test

The majority of calves that participated in the suckling reflex test displayed a suckling reflex with each type of teat that was tested (control: 47 out of 57 calves; odor: 45 out of 57 calves; taste: 45 out of 57 calves). Thirty-four out of 57 calves performed the “licking lips” behavior when exposed to the "Freshly Cut Grass" odor through the mesh of the test apparatus, during the presentation of the odor teat. In contrast, none of the calves performed this behavior when presented with the control teat and the taste teat. Thus, the proportion of calves performing the “licking lips” behavior when exposed to the “Freshly Cut Grass” odor (34/57) was significantly higher compared to the proportion of calves performing this behavior when exposed to the control teat (0/57) and the taste teat (0/57) (χ^2^ = 27.648, df = 1, *p* < 0.001).

### Odor test

#### Order of teat investigation

**Fig. 4:**
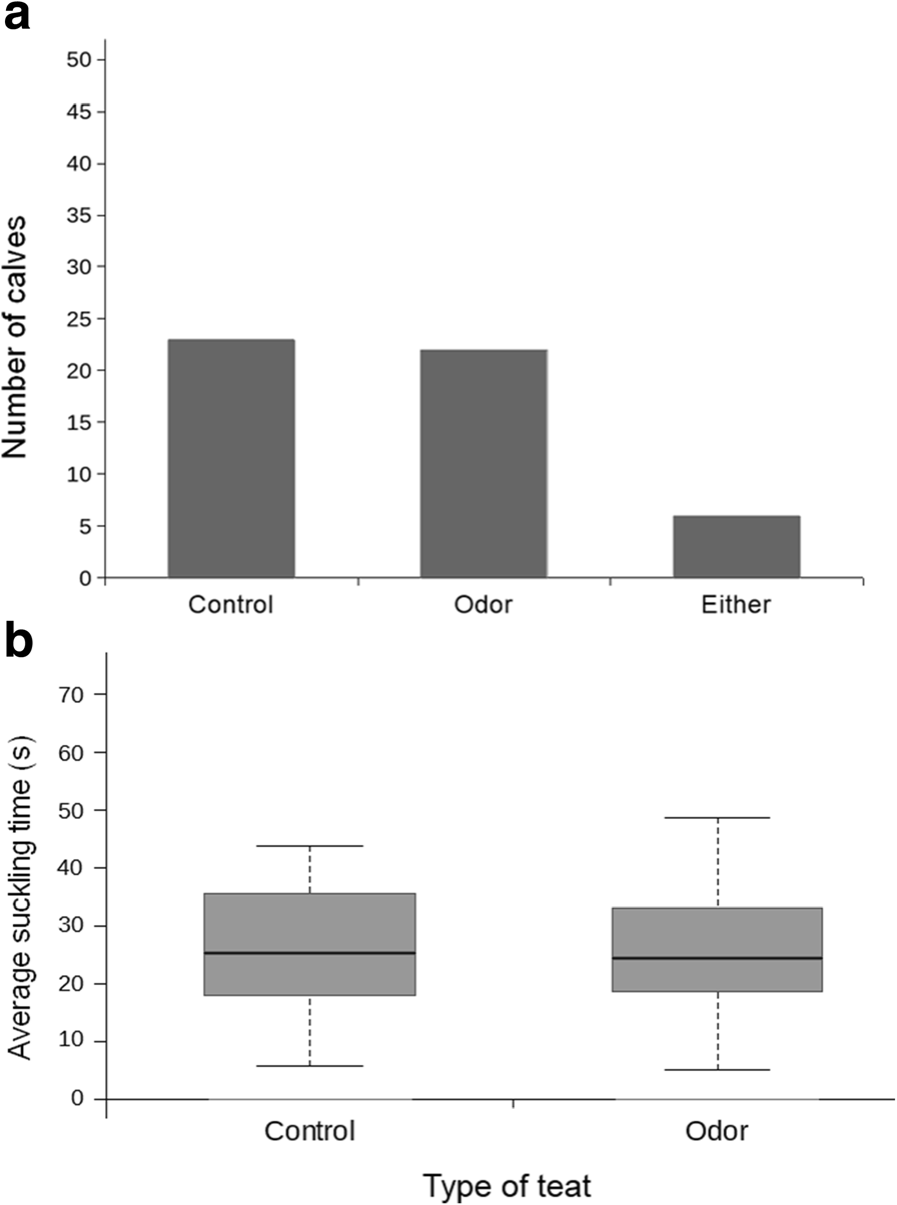
Order of teat investigation and median suckling times in the odor test. **a**. Number of calves (*n* = 51) that investigated a particular type of teat as the first one in the majority of their testing sessions in the odor test; **b**. Median suckling time (s) for the control teat (*n* = 51, median = 25.38, IQR = 18.12–35.50) and the odor teat (*n* = 51, median = 24.88, IQR = 19.14–33.69) in the odor test

Twenty-three out of 51 calves investigated the control teat first in the majority of testing sessions while 22 calves investigated the odor teat first (Fig. 4, Panel a). Six out of 51 calves investigated either teat first for an equal number of sessions (Fig. 4, Panel a). The calves did not show a significant preference regarding the order of teat investigation in the odor test (Binomial: *n* = 45, 23:22, *p* = 1).

#### Suckling times

When assessing the suckling times for the control teat versus the odor teat, the calves did not show a significant preference for any of the two teats in the odor test (Wilcoxon: *n* = 51, Z = − 0.272, *p* = 0.786). The median suckling time was 25.38 (IQR = 18.12–35.50) s for the control teat and 24.88 (IQR = 19.14–33.69) s for the odor teat (Fig. 4, Panel b).

### Taste test

#### Order of teat investigation

**Fig. 5:**
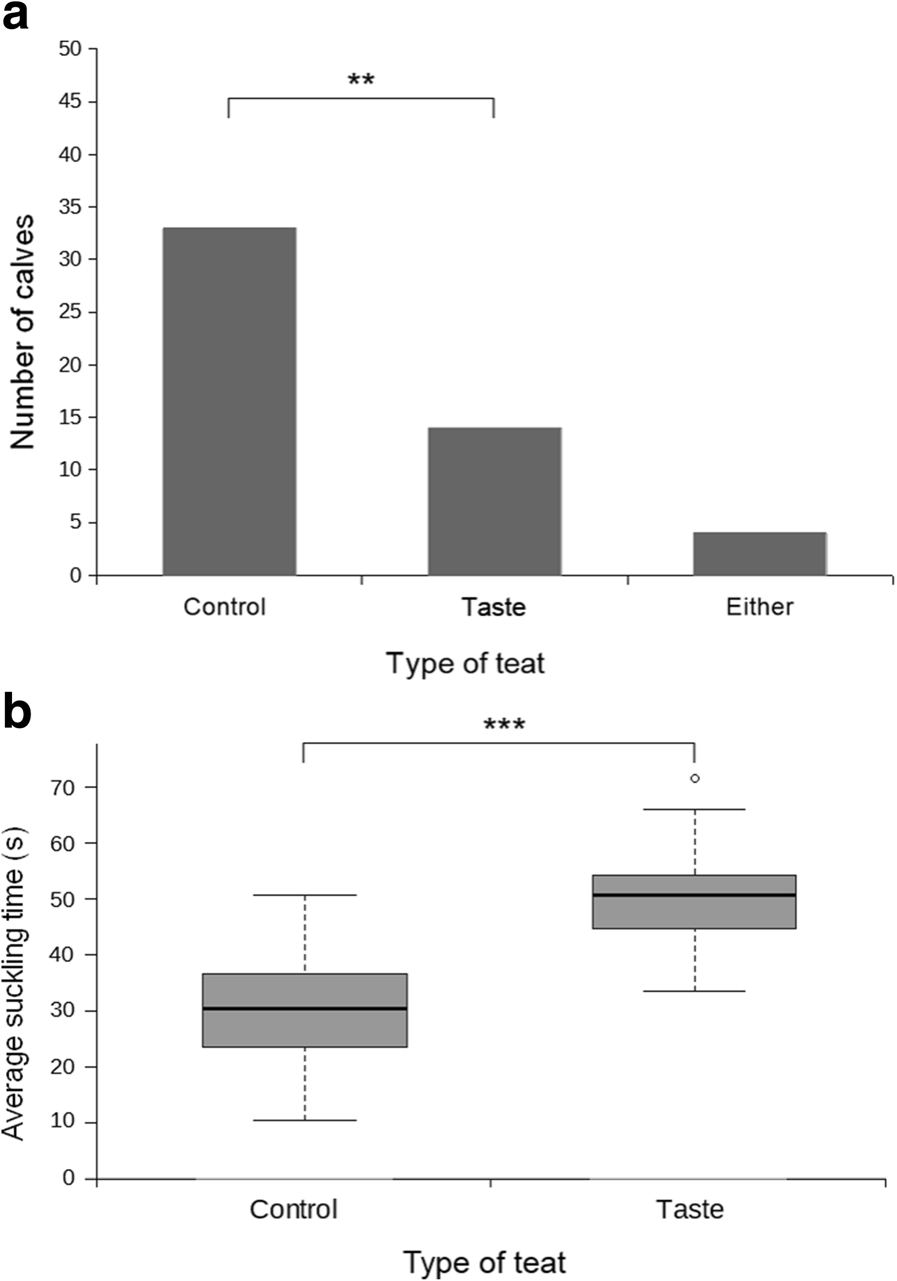
Order of teat investigation and median suckling times in the taste test. **a**. Number of calves (n = 51) that investigated a particular type of teat as the first one in the majority of their testing sessions in the taste test. Asterisks (**) correspond to *p* < 0.01; **b**. Median suckling time (s) for the control teat (n = 51, median = 30.56, IQR = 23.48–36.71) and the taste teat (n = 51, median = 50.67, IQR = 44.71–54.34) in the taste test. Asterisks (***) correspond to *p* < 0.001

Thirty-three out of 51 calves investigated the control teat first in the majority of testing sessions while 14 calves investigated the taste teat first (Fig. 5, Panel a). Four out of 51 calves investigated either teat first for an equal number of sessions (Fig. 5, Panel a). The calves showed a significant preference for the control teat regarding the order of teat investigation in the taste test (Binomial: *n* = 47, 33:14, *p* = 0.008).

#### Suckling times

When assessing the suckling times for the control teat versus the taste teat, the calves showed a significant preference for suckling the taste teat in the taste test (Wilcoxon: *n* = 51, Z = − 6.177, *p* < 0.001). The median suckling time was 30.56 (IQR = 23.48–36.71) s for the control teat and 50.67 (IQR = 44.71–54.34) s for the taste teat (Fig. 5, Panel b).

#### Comparison of total suckling times in the odor test versus the taste test

**Fig. 6:**
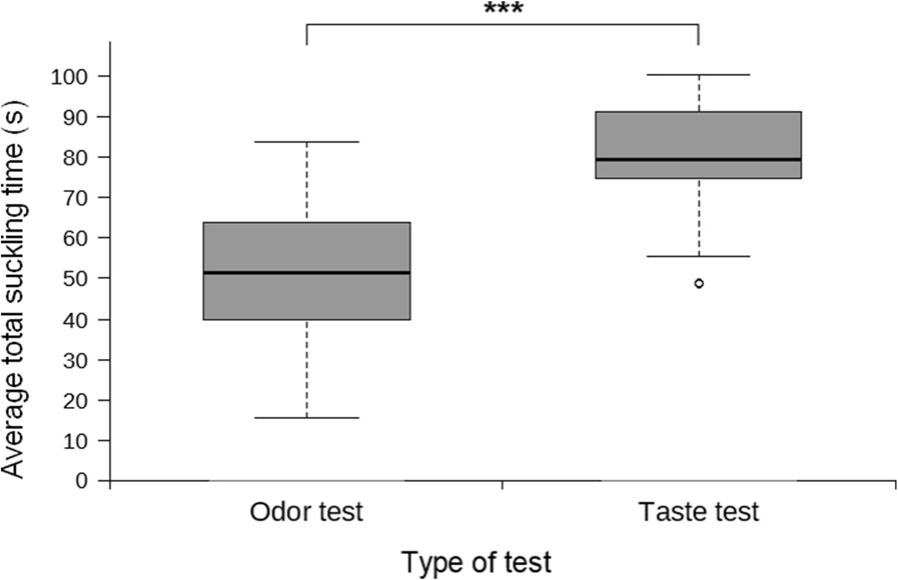
Comparison of total suckling times in the odor test versus the taste test. Median total suckling time (s) for the odor test (*n* = 51, median = 51.25, IQR = 39.80–63.38) versus the taste test (*n* = 51, median = 79.29, IQR = 74.70–91.32). Asterisks (***) correspond to *p* < 0.001

When comparing the total suckling times (ST_T_ = ST_C_ + ST_M_) between the odor test and the taste test, the calves showed a significantly longer total suckling time during the taste test (median = 79.29 s, IQR = 74.70–91.32 s) compared to the odor test (media*n* = 51.25 s, IQR = 39.80–63.38 s) (Wilcoxon: n = 51, Z = − 6.215, *p* < 0.001; Fig. 6).

#### Differences in suckling times between sexes

**Fig. 7:**
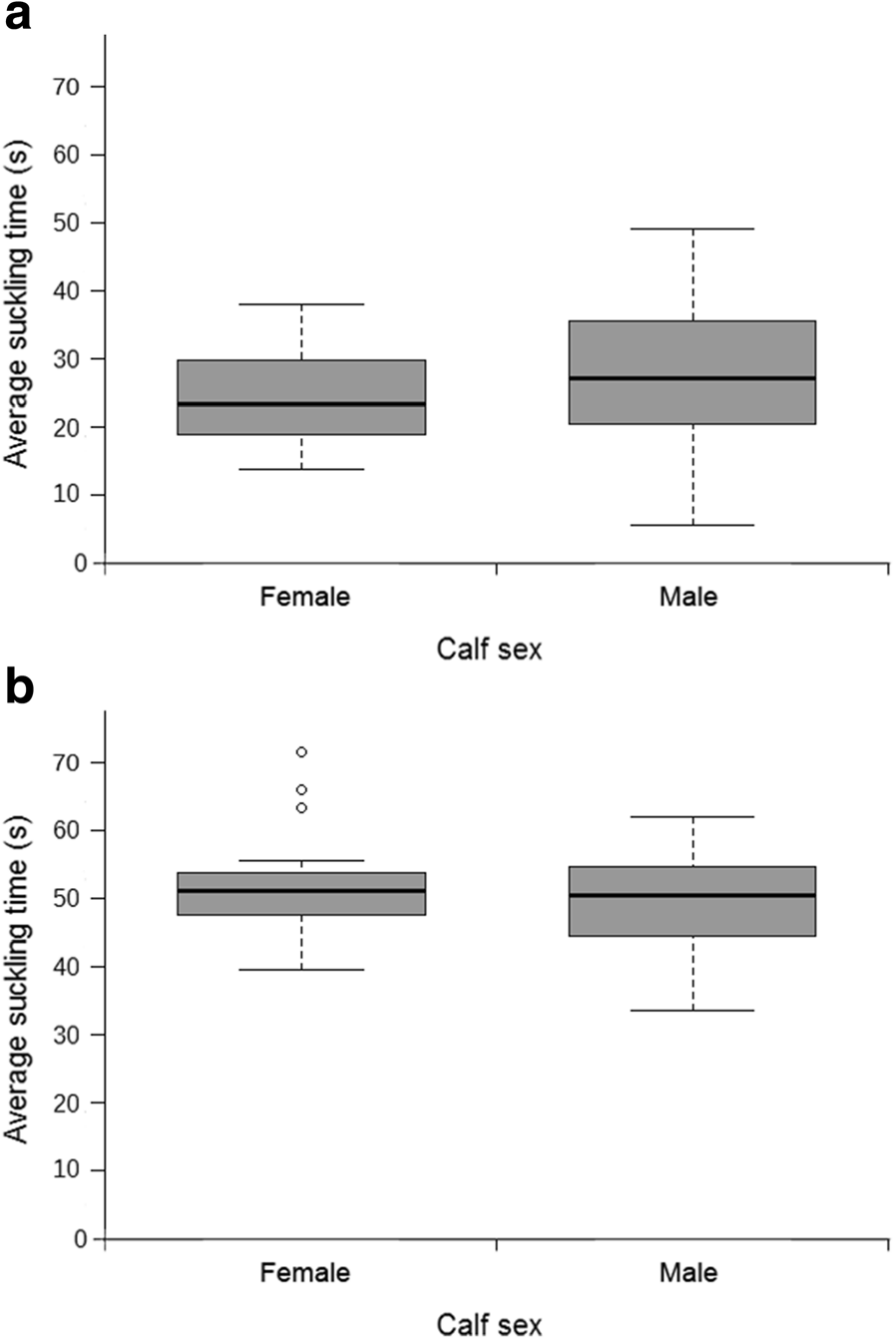
Differences in suckling times between sexes. Median suckling times for the odor and taste teats in A. Median suckling time for the odor teat in male (*n* = 29, median = 27.22, IQR = 20.50–35.56) and female (*n* = 22, median = 23.44, IQR = 19.07–29.33) calves. B. Median suckling time for the taste teat in male (*n* = 29, median = 50.57, IQR = 44.44–54.70) and female (*n* = 22, median = 51.22, IQR = 47.69–53.49) calves

No significant differences were found in the average suckling times for the modified teat between male and female calves. This was true both for the odor test (Mann-Whitney: *n* = 51, *U* = 260.5, *p* = 0.27; Fig. 7, Panel a) and for the taste test (Mann-Whitney: *n* = 51, *U* = 336, *p* = 0.754; Fig. 7, Panel b), respectively.

## Discussion

The majority of calves in this study were able to stand up voluntarily and suckle from an artificial teat from the second or third day of age on. The odor stimulus elicited a “licking lips” behavioral response during the suckling reflex test that was not observed during the presentation of the control teat and the glucose-coated teat. However, this odor stimulus had no significant effect on the preference of the calves when first investigating or suckling an artificial teat. In the taste test, the majority of the animals showed a significant preference for investigating the control teat first and for suckling the glucose-coated teat. Furthermore, the calves showed a significantly longer total suckling time in the taste test compared to the odor test. There were no significant differences between male and female calves with regard to suckling times.

### Readiness of newborn calves to suckle an artificial teat

Stafford et al. [7] reported that the majority of newborn calves were able to efficiently suckle an artificial teat without assistance by the second day of age. In accordance with these findings, the majority of the calves in our study were able to stand up voluntarily and suckle the artificial teat for the first time from the second or third day of age on. However, only very few calves managed to stand up voluntarily and suckle on the first day of age and about 13% of the calves in the present study needed as many as four or five days before they succeeded in the task. While this percentage is not particularly high, it might still signify a problem for the farm and the welfare of the calves in terms of potential losses in weight gain, poor function of the immune system and extra labor dedicated to calf care.

Furthermore, the majority of the calves that were not motivated to stand up voluntarily and suckle when attempting to stimulate their suckling reflex with a finger presented a suckling reflex when a teat (plain or modified with an odor or taste substance) was inserted in their mouth. This raises the question of whether a number of healthy newborn calves have difficulty in standing up and successfully suckling the artificial teat not primarily because of its properties but because of a general lack of natural sensory cues that motivate the calf to stand up and search for a teat.

### Responses to the odor substance

#### Suckling reflex test

Sixty percent of the calves responded with a “licking lips” behavior when smelling the "Freshly Cut Grass" odor through the mesh of the test apparatus during the suckling reflex test. This behavioral response was immediate and consistent among the calves that performed it and was only elicited in the presence of the tested odor. The control teat and the taste teat failed to elicit this or other behavioral responses upon teat presentation. The “licking lips” behavior greatly resembled a rapid gustatory response which was neither attractive nor aversive according to the description for other mammalian species [26, 27]. Taking into consideration that the calves are exposed daily to volatile compounds from grass feed via the milk they are fed [22–24], it is possible that the animals perceived the odor as a property of a potentially edible substance.

#### Odor test

Despite the observation of a “licking lips” behavioral response in the suckling reflex test, the calves did not show a significant preference for a teat (control or odor) in the odor test neither with regard to the order of teat investigation nor in terms of suckling times. These results indicate that the selected odor had no significant effect on the preference of the calves when suckling an artificial teat, and support the notion that the tested odor was perceived as neither attractive nor aversive by the calves participating in the odor test.

Several reasons may explain the lack of an effect of the "Freshly Cut Grass" odor on the teat preference of the calves. Being a synthetic fragrance oil, the selected odor may not have imitated the natural odor of freshly cut grass to such a degree that it would be clearly recognizable as a feed-related odor. Young calves are not introduced to grass feeds before two weeks of age on average [21] and while volatile compounds of grass feed are present in the whole milk fed to the newborn animals [22–24], these grass-related odors might be too subtle to be readily distinguished as feed by the calves, more so if the animals are presented with an odor that resembles the original only to some degree. Regarding the order of teat investigation, Hafez and Lineweaver [1] claimed that newborn calves rely primarily on their senses of touch and vision to locate a teat when suckling a dam. This observation might explain why the calves in the present study did not show a preference for specifically locating the teat with the odor cue first.

Another way to potentially enhance the calf’s preference for a specific odor would have been to apply the odor substance on the udder of the dam a few hours before labor and until separation from the calf. Newborn animals are able to become attracted to odors that are present on the skin of the mammary glands of the mother during suckling [28–30]. As the calves in our study spent at least a few hours with the dam before separation and were allowed to suckle during that period, a consistent presence of the selected odor on the udder of the dam might have led to a significant preference for that odor and the respective artificial teat in the odor test. In the case of an essential or fragrance oil used as the odor stimulus, such application on the udder could be achieved quickly, efficiently and economically in the form of spray or ointment.

### Responses to the taste substance

#### Suckling reflex test and order of teat investigation in the taste test

The calves showed a significant preference for investigating the control teat first in the taste test, indicating that the presence of glucose had an effect on the calves’ choice regarding order of teat investigation. It is likely that the calves visually distinguished between the orange-colored control teat and the white-colored (due to the glucose powder) modified teat used in the taste test. Phillips and Lomas [31] reported that cattle are capable of distinguishing colors particularly between orange, yellow and red, while Phillips and Weiguo [32] suggested that calves can also differentiate items of various light intensities. Furthermore, as mentioned previously, Hafez and Lineweaver [1] suggested that newborn calves will attempt to locate a teat primarily using their senses of touch and vision. Assuming that the calves could visually distinguish between the two teats, a possible explanation would be that the calves initially avoided the glucose-coated teat due to food neophobia. This behavioral reaction has been observed in cattle [33–35] and is described as the reluctance of sampling new foods for fear that they may be toxic or poisonous [36].

To our knowledge, it is not known if cattle can detect the odor of glucose so it is unlikely that an unintentional odor cue affected the order of teat investigation of the calves in this test. This notion is further supported by the lack of behavioral responses when presenting the calves with the glucose-coated teat during the suckling reflex test, which was in accordance with the lack of responses to the control teat but in contrast to the “licking lips” response observed when presented with the "Freshly Cut Grass" odor during the same test.

#### Suckling preference

Contrary to the order of investigation analysis, the calves showed a significant preference for suckling the glucose-coated teat in the taste test. Additionally, the average suckling time for the taste teat was nearly twice as long as the average suckling time for the control teat. These findings are in agreement with the established preference of calves for sweet-tasting substances, including glucose [17, 37]. Additionally, de Passillé and Rushen [20] showed that the carbohydrates in the milk were primarily responsible for eliciting non-nutritive suckling following a brief episode of suckling milk in calves of 4–18 weeks of age, further underlining the ability of glucose to stimulate suckling.

#### Effect of the odor and the taste substances on total suckling time

An important consideration when assessing suckling times per specific teat (control teat, modified teat) is the natural tendency of calves to switch between teats during suckling [38, 39]. When suckling the dam, the calf will suckle from more than one teat and rapidly switch between teats during a single feeding bout [1, 38, 39]. Factors such as reduced milk flow and increased teat firmness can cause the calf to perform these teat switches several times during suckling [38, 39].

We, too, observed that the calves in the present study performed these switches between the two teats during the testing sessions, both in the odor and the taste test. Thus, we considered that it would be useful to examine if either the taste or the odor stimulus motivated the calves to suckle for a longer period of time *in total* during their testing sessions in comparison to the other stimulus. The calves in the present study showed a significantly longer total suckling time in the taste test compared to the odor test. In accordance with the notion of de Passillé and Rushen [20] regarding the effect of carbohydrates found in milk on the stimulation of non-nutritive suckling in calves as old as four weeks of age, our results demonstrate that calves up to two weeks of age displayed a significantly increased motivation for non-nutritive suckling when presented with a sweet-tasting substance but not when presented with the "Freshly Cut Grass" odor.

The median total suckling times differed significantly between the odor test (median = 51.25 s, IQR = 39.80–63.38 s) and the taste test (median = 79.29 s, IQR = 74.70–91.32 s). Lidfors et al. [40] reported that, on the first day of age, the suckling activity of beef calves accounted for the 57.8% - 85.2% of the total time of a feeding bout, with the rest of the time commonly spent in butting, switching between teats, pausing activity or repositioning. Similarly, Appleby et al. [41] found that dairy calves of approximately 25 days of age spent about 80% of time suckling the artificial teat during the first feeding bout of the day. Considering that our testing sessions lasted for two minutes, the calves in the odor test spent on average 43% of a testing session suckling a teat, while in the taste test they spent 66% of a testing session on suckling. This suggests that the presence of glucose rather than the "Freshly Cut Grass" odor elicited suckling bouts that came closer to the natural average suckling time. It is also likely that an even longer total suckling time could be achieved for the taste test provided that the substance would not wear off as quickly due to suckling.

#### Differences between sexes

There were no significant differences between male and female calves regarding suckling times in the present study for both the odor test and the taste test. To our knowledge, there are no studies so far regarding sex differences in newborn mammals in relation to the perception of taste and odor. Such differences in chemosensory perception between sexes have been reported for some adult mammals [42–44] and seem to be mostly caused by the effects of sex hormones. Cattle reach puberty at about six months of age [3], hence the effects of sex hormones on the calves in the present study regarding the senses of smell and taste cannot be considered, and it is unlikely that sex differentiation for these senses would be beneficial in newborn animals in any behavioral context.

#### Potential applications in the dairy industry

Glucose, which had a significant effect on the teat preference and suckling motivation of the calves in this study, is an inexpensive substance that is commonly used at dairy farms for the preparation of electrolyte solutions for calves. Coating an artificial teat with glucose is a quick and simple task that should not unduly burden the workload of the calf caretaker during feeding times. While further research is required in order to examine how different substances might facilitate the learning process of using an artificial teat in newborn calves, glucose applied on the artificial teat may be able to increase suckling motivation in calves that require frequent stimulation of the suckling reflex from the staff in order to consume a full milk meal, or in calves that have low motivation to suckle due to illness. This, in turn, not only improves the welfare of the calves but may also benefit the dairy farmer’s economy by decreasing the caretakers’ workload when dealing with a number of newborn calves requiring assistance during feeding, and by potentially preventing undernutrition and weight loss in calves that have difficulty consuming a full milk meal without assistance.

## Conclusions

The results of the present study show that the taste of glucose, but not the "Freshly Cut Grass" odor, had a significant effect on the calves’ teat preference when suckling a dry artificial teat. More importantly, the results show that glucose significantly increased the motivation of newborn calves to suckle a dry artificial teat. No differences in suckling preference were observed between the two sexes.

## Additional file

Additional file 1: Survey among Swedish caretakers on the use of an artificial teat in newborn dairy calves. The file presents data regarding the background, questionnaire, results and conclusion of an online survey conducted in October 2017 and targeting Swedish caretakers and their experiences at work on the use of an artificial teat in newborn dairy calves. (DOCX 16 kb)

## Abbreviations

ST_C_: Suckling time at control teat
ST_M_: Suckling time at modified teat
ST_T_: Total suckling time (ST_T_ = ST_C_ + ST_M_)
