# Supplementary material for: Effects of an odor or taste stimulus applied to an artificial teat on the suckling behavior of newborn dairy calves
Source: J Anim Sci Technol. 2018 Apr 16;60:16. doi: 10.1186/s40781-018-0164-x (PMC6006940; doi:10.1186/s40781-018-0164-x)
Supplement: Supplementary file 1 — Survey among Swedish caretakers on the use of an artificial teat in newborn dairy calves. The file presents data regarding the background, questionnaire, results and conclusion of an online survey conducted in October 2017 and targeting Swedish caretakers and their experiences at work on the use of an artificial teat in newborn dairy calves. (DOCX 16 kb) [file 40781_2018_164_MOESM1_ESM.docx]

**Survey among Swedish caretakers on the use of an artificial teat**

**in newborn dairy calves**

**Background and method**

Anecdotal data from calf caretakers and dairy farm owners have suggested that a number of otherwise healthy newborn dairy calves have difficulty using an artificial teat during the first 1-3 days of life, and that this problem persists even when using an artificial teat that is dipped in or releases milk. To our knowledge, there is currently no published study that presents data on this topic. In October 2017, we initiated a preliminary online survey on Google Forms that we made available to calf caretakers and dairy farmers in Sweden via Facebook. The survey is composed of an introductory text and three questions that must be answered (obligatory fields), with only one response per question allowed to be selected. The survey is open until December 31st 2017 and is available at the following link, in Swedish: <https://goo.gl/forms/dWhgCTHjkBctAYBw1>. The survey is anonymous - no personal data on the participants has been registered by Google Forms that is visible or available to the authors.

The introductory text follows in English:

"Hello! My name is Maria Malidaki and I am a veterinarian who researches the behavior of dairy cows and calves.

A number of newborn and otherwise healthy calves have difficulty or entirely refuse to use the artificial teat attached to a teat bucket during the first 1-3 days after birth. My personal experience along with discussions with caretakers indicates that such calves do not become motivated to use the artificial teat even when the teat is dipped in or releases milk. Often, the calves become very hungry before they start using the artificial teat effectively. Do you perhaps recognize this issue?

As a part of my research I would kindly like to collect preliminary information on the problem directly from our farmers and caretakers. It only takes a couple of minutes to answer 2 simple questions! I ask for your help in order to better understand the problem and research a solution for it.

You are welcome to participate in the survey until December 31st 2017. Feel free to share with other dairy farmers and caretakers that might be interested!

Thank you for your time and help!"

**Questionnaire and results**

The questionnaire is as follows, in English. The survey received responses from 55 individuals as of October 31st 2017.

**Question 1**

It happens that a number of newborn and otherwise healthy calves refuse to use the artificial teat for 1-3 days after birth:

- **0 (0%)** responded: It never happens to my newborn dairy calves.
- **14 (25.5%)** responded: It can happen, but very rarely. It is not really a problem for me.
- **28 (50.9%)** responded: It can happen to some calves, but I do not see it as a problem.
- **13 (23.6%)** responded: It can happen to some calves, and when it happens I see it as a problem.

**Question 2**

The newborn calves that refuse to use the artificial teat become immediately motivated and use the teat without a problem after I have dipped it in milk / if it releases milk in the calf's mouth:

- **1 (1.8%)** responded: My newborn calves never have problems using the artificial teat.
- **31 (56.4%)** responded: Some calves can be motivated and some keep refusing the artificial teat for some time.
- **6 (10.9%)** responded: Yes, they become immediately motivated and the problem is solved in this way.
- **17 (30.9%)** responded: No, they do not become motivated and continue refusing the artificial teat. They do not drink from it before they have missed a number of meals.

**Question 3**

How many meals on average do the newborn calves that refuse the artificial teat miss until they start using it effectively? (Attention: force-feeding with an esophageal tube does not count as a meal in this survey):

- **2 (3.6%)** responded: My newborn calves never have problems using the artificial teat and thus never miss a meal because of it.
- **39 (70.9%)** responded: They can miss 1-2 meals before they start using the artificial teat. I feed them with an esophageal tube or wait until they have started drinking themselves.
- **12 (21.8%)** responded: They can miss 3-4 meals before they start using the artificial teat. I feed them with an esophageal tube or wait until they have started drinking themselves.
- **2 (3.6%)** responded: They can miss up to 6 meals before they start using the artificial teat. I feed them with an esophageal tube or wait until they have started drinking themselves.

**Current conclusions**

The majority of the participants (74.5%) responded that they recognize the stated problem in a number of newborn dairy calves. However, only 13 out of 55 participants feel that this actually is a problematic situation. This is perhaps affected by the fact that calves do not tend to miss more than two meals before they start using the artificial teat, as the majority of participants (70.9%) stated. It is interesting to note that the majority (87.3%) responded that a number of calves do not get motivated in using the artificial teat despite it being dipped in or releasing milk in the mouth of the calves. These preliminary results suggest that the taste of milk alone is not always enough in eliciting successful suckling of an artificial teat in newborn dairy calves during the first 72 hours after birth. Other factors that might influence successful suckling from an artificial teat during the first days of life are open to research.
